# Ecological functions of fungal sesquiterpenes in the food preference and fitness of soil Collembola

**DOI:** 10.1098/rsos.231549

**Published:** 2024-02-21

**Authors:** Matthäus Slonka, Ilka Vosteen, Artemio Mendoza-Mendoza, Michael Rostás

**Affiliations:** ^1^ Agricultural Entomology, Department of Crop Sciences, University of Göttingen, Grisebachstr. 6, 37077 Göttingen, Germany; ^2^ Faculty of Agriculture and Life Sciences, Department of Wine, Food and Molecular Biosciences, Lincoln University, Lincoln, New Zealand

**Keywords:** fungivory, *Folsomia candida*, olfaction, springtails, *Trichoderma* *virens*, volatile organic compounds

## Abstract

Volatile organic compounds (VOCs) emitted by fungi play a key role in locating and selecting hosts for fungivorous arthropods. However, the ecological functions of many common VOC classes, such as sesquiterpenes, remain unknown. Mutants of *Trichoderma virens*, defective in the emission of most sesquiterpenes owing to the deletion of the terpene cyclase *vir4*, were used to evaluate the role of this compound class in the food preference and fitness of the soil Collembola *Folsomia candida.* Choice experiments with and without direct contact with fungal mycelium revealed that Collembola were preferentially attracted to *Δvir4* mutants impaired in sesquiterpene synthesis compared to wild-type *T. virens*. Grazing by *F. candida* on the sesquiterpene deficient *T. virens* strain had no effect on Collembola survival, reproduction and growth compared to wild-type *T. virens*. The results suggest that sesquiterpenes play an important role in fungal defence as repellents, but not as deterrents or toxins, against fungivorous Collembola. Our research contributes to the understanding of ecological interactions between fungi and fungivorous arthropods, providing insights into the specific ecological functions of sesquiterpenes. The study has implications for chemical ecology and the dynamics of multitrophic interactions in soil ecosystems.

## 1. Introduction

The mycelia of soil fungi are constantly challenged by predation from fungivorous arthropods like Collembola which discriminate between fungal strains and display marked preferences [1]. Selective grazing by fungivores can change fungal community composition [2] and shape the outcome of competitive interactions between different fungi, thus increasing the competitiveness of some strains [3]. Fungivore grazing can both limit the growth of dominant species and stimulate less competitive species, leading to the reversal of competitive hierarchies [4]. On the other hand, fungal succession can be accelerated if less competitive strains are preferentially grazed [5,6].

Several factors, such as nutrient content and secondary metabolite production, determine the suitability of fungi as food for fungivores [7,8]. Fungal volatile organic compounds (VOCs) serve as important cues for mycelial location and selection by soil fungivores, but may also act as repellents [9]. VOCs are small (less than 300 Da) secondary metabolites that readily diffuse through air and water in the soil. Because of these properties, they are considered ideal infochemicals for long-range communication. Soil microorganisms such as fungi and bacteria produce a highly diverse spectrum of VOCs belonging to various chemical classes, as they rely mostly on chemical signalling to exchange information with their environment [10,11]. However, the ecological function of many identified compounds has not been established yet [12,13].

Staaden *et al*. [14] showed that three Collembola species could discriminate between fungal species on the basis of odour alone. However, the compounds responsible were not identified in that publication. In fact, few compounds have been identified that elicit responses in Collembola foraging behaviour [15–18]. Typically, eight-carbon compounds such as 1-octen-3-ol are prominent in fungal odours and are known to be involved in the chemotaxis of Collembola and other fungivores [9].

*Trichoderma* (teleomorph: Hypocrea) is a genus of filamentous ascomycete fungi that is consistently avoided by Collembola. This is consistent with the fact that Collembola generally display poor fitness parameters like reproduction and survival when feeding on *Trichoderma* species, compared to other fungi [19–22]. *Trichoderma* spp. are ubiquitous soil fungi with mycoparasitic and saprophytic lifestyles. Some *Trichoderma* spp. also colonize roots as endophytes where they enhance plant growth and stress resistance [23,24]. *Trichoderma* effectors involved in plant interactions are well studied and include enzymes, peptides, non-volatile secondary metabolites and VOCs [25,26]. In a multitrophic context, VOCs emitted by *Trichoderma atroviride* have even been found to induce plant resistance and to inhibit herbivory on aboveground plant parts [27]. However, there is little information on the role of *Trichoderma* VOCs in the interaction with belowground fungivores.

The composition of the volatile bouquet emitted by *Trichoderma* is species- and even strain-specific. Consequently, differences in VOC emission between strains of *Trichoderma* may account for differences in their efficacy against pathogens and pests [28]. In many cases, the success of *Trichoderma* depends on its ability to rapidly outcompete plant pathogens in the rhizosphere, a process in which VOCs are known to play an important role. Certain VOCs may also act as foraging cues for fungivores, potentially disrupting these competitive interactions between fungi by grazing on the hyphae [2–6]. Bridging the knowledge gaps regarding the impact of VOCs on interactions between *Trichoderma* and fungivores is crucial for understanding the central role of volatiles in multitrophic interactions in the soil.

In this research, we studied the role of *Trichoderma virens* volatiles in its interaction with the fungivorous Collembola *Folsomia candida* Willem. Unlike many other *Trichoderma* species, the volatile profile of *T. virens* lacks well-described components such as 1-octen-3-ol or 6-pentyl-α-pyrone. Instead, the odour emitted by *T. virens* mostly consists mainly of different sesquiterpenes [29,30]. The precise biological functions of sesquiterpenes in fungal-fungivore interactions remain largely unknown. While sesquiterpenes are known to be repellent for herbivores and attractive to their natural enemies when emitted by plants, it has been postulated that they may play a defensive role for fungi against their predators [31,32]. However, a small number of studies also suggest that microbial sesquiterpenes can be attractive for fungivorous Collembola [15] and beetles [33].

Most *T. virens* volatile terpenes are produced by a single terpene synthase (*vir4*) located in the *vir* cluster [29]. To specifically test the role of several *T. virens* sesquiterpenes in fungal-fungivore interactions, we used a *vir4* deletion mutant of *T. virens* that was deficient in the production of sesquiterpene volatiles. We hypothesized that the abundant constitutive emission of fungal sesquiterpenes serves as an allomone against predators. The results of our study indicate that fungal sesquiterpenes act as repellents against *F. candida*, but do not possess feeding deterrent or toxic properties.

## 2. Material and methods

### 2.1. Organisms

Volatile-deficient mutants of *T. virens* Gv29.8, previously generated in a separate study, were used [34]. The terpene cyclase *vir4* had been deleted from Gv29.8 (hereafter called wild-type) to produce the knock-out strain Gv29.8Δ*vir4* (hereafter called knock-out). In this knock-out, a copy of *vir4* was randomly inserted into the genome, creating the complementation strain Gv29.8Δ*vir4::vir4* (hereafter called complement).

*Trichoderma virens* strains were cultivated on potato dextrose agar (PDA; Carl Roth GmbH & Co. KG, Karlsruhe, Germany, prepared according to manufacturer's instructions) at 25°C. Mycelium plugs were extracted from the outer edge of 7-day-old colonies using a cork borer. *Folsomia candida* was reared in Petri dishes on a layer of plaster of Paris mixed with activated charcoal (13 : 1, w/w) at 20°C in darkness. Collembola were fed a diet of dry baker's yeast (Lucullus Food Service GmbH & Co. KG, Trittau, Germany) on a weekly base, provided ad libitum. During feeding, the Petri dishes were aerated. Tap water was added when needed to maintain humidity levels. Before being used in the experiments, Collembola were transferred to a new Petri plate with pure plaster of Paris and starved for 48 h.

### 2.2. Analysis of fungal volatile organic compounds

Petri dishes (Ø = 35 mm) were filled with 4 ml of PDA and inoculated with *T. virens* strains. The plates were cultivated at 25°C in darkness. After 3 days, the lid was removed, and the plate was transferred into a top-open headspace glass vessel (Ø = 50 mm, height = 115 mm). The vessel had inlet and outlet openings for airflow on the sides (opening Ø = 9 mm). The inlets were closed with screw caps, and the upper opening was sealed with polyester oven bag foil (Confresco Frischhalteprodukte GmbH & Co. KG, Minden, Germany). The glass vessel was then further incubated for 24 h at 25°C in darkness. After incubation, the vessel was connected to an air inflow adjusted to 320 ml purified air min^−1^, and the air was sucked out at a rate of 300 ml min^−1^ with a Laboport vacuum pump (KNF Neuberger GmbH, Freiburg, Germany). Before entering the headspace of the vessel, the air passed through two hydrocarbon/moisture traps (Agilent Technologies, Model HT200-4) for filtration. Headspace sampling was carried out for 2 h in darkness at 24°C and 45% relative humidity. Volatiles were collected with porapak Q traps (VCT-1/4-3-POR-Q, www.ars-fla.com). Volatile traps were placed in the outflow opening and eluted with 150 µl dichloromethane. The traps were rinsed with 1 ml dichloromethane before each sampling. Empty Petri dishes and Petri dishes filled with PDA were analysed similarly to identify background volatiles. For each treatment, five replicates were analysed. Volatile collection was performed with six samples at a time, and this process was repeated six times within 6 days.

We added 200 ng of tetralin (20 ng µl^−1^, Sigma-Aldrich Chemie GmbH, Taufkirchen, Germany) as an internal standard to each sample. Then, 40 µl of the sample was transferred to a new glass vial with an insert glass for gas chromatography-mass spectrometry (GC-MS) analysis using an Agilent 5977B GC/MSD. An HP5-MS analytical column was used (30 m × 0.25 mm inner diameter, 0.25 µm film thickness, Agilent Technologies, Santa Clara, CA, USA). Two microlitres of the eluate were injected in splitless mode. The injection temperature was set to 220°C, and the pressure was maintained at 18.84 psi. The oven temperature was held at 40°C for 3 min and then raised to 320°C at 8°C min^−1^ and held at this temperature for 8 min. Helium was used as carrier gas at a constant flux of 1.5 ml min^−1^.

Chromatogram analysis was performed with the software Enhanced ChemStation, MSD ChemStation F.01.03.2357. VOCs were tentatively identified by comparing mass spectra and retention indices (RI) with literature values available on the NIST website (National Institute of Standards and Technology, USA, www.webbook.nist.gov). When applicable, VOCs were also compared with cannabis oil terpene standards to confirm their identity (Agilent Technologies, Santa Clara, CA, USA). Compounds were quantified by comparing their peak areas with the area of the internal tetralin standard.

### 2.3. Analysis of fungal CO_2_ emission

Fungal colonies were cultivated in Petri dishes (Ø = 35 mm) for 4 days as previously described. One hour before analysis, colonies were enclosed in the same glass vessel used for VOC analysis to allow for the build-up of a CO_2_ gradient. A CARBOCAP GM70 (Vaisala, Helsinki, Finnland) device was used for CO_2_ measurements in the following manner: the probe was allowed to adjust to background CO_2_ levels and was then inserted into one side of the source vessel for 4 min. The maximum peak value was noted down. After completing the measurement, the probe was retracted and allowed to adjust to atmospheric levels. For each treatment, 15 samples were measured. To determine mycelial weight, the mycelium was first separated from the agar. We placed the overgrown agar in a beaker of water and heated it in a microwave until the agar melted and dissolved in the water. The mycelium was then freeze-dried and weighed. The recorded CO_2_ levels were normalized to the mycelial weight.

### 2.4. Olfactometer test

To assess the orientation of *F. candida* towards volatiles emitted by the fungal strains, a modified version of compartmented olfactometers, following the method described by Staaden *et al*. [14], was used. Briefly, two agar plug cut-outs (Ø = 20 mm) from the fungal strains of interest were placed in a four-compartment Petri dish (Hedinger, Type 639102) as odour sources. One compartment contained only PDA, another compartment was left empty, serving as a control. A hardened filter paper (Carl Roth, Type 1505/NL31.1) was placed on top and held in place with a bottomless Petri dish (BIOLOGIX, Type 66–1501). The position of the compartments of the Petri dish below were marked on the filter paper arena with a lead pencil and the paper was moistened with 1 ml of water to provide *F. candida* with the necessary humidity. Thirty adult Collembola were released onto the filter paper. The olfactometer was closed with a lid and sealed with Parafilm. We tested the response of *F. candida* towards three combinations of *T. virens* strains: wild-type versus wild-type, wild-type versus knock-out, and wild-type versus complement. Olfactometers with different strain combinations were placed in separate metal boxes. The boxes were lined with moist paper to maintain a humid atmosphere necessary fo the survival of *F. candida*. The boxes were then kept at 21°C in darkness. Springtail location in relation to the compartments of the bottom Petri dish was observed at 0.5 h, 1 h, 2 h, 4 h, 8 h, 12 h, 24 h, 32 h, 48 h, 56 h and 72 h after release and documented by photography using a Panasonic Lumix DMC-LX7 digital camera. Additional water was added every 12 h to maintain sufficient humidity in the walking arena. Eight olfactometers were used per strain combination in each experimental round, and the experiment was repeated three times, resulting in 24 replicates for each strain combination.

### 2.5. No-choice acceptance test

Agar plugs (Ø = 9 mm) with or without *T. virens* strains were placed in the centre of a Petri dish (Ø = 92 mm) filled with moist plaster of Paris. There were 10 replications for each treatment and the experiment was repeated twice in total, resulting in 20 replications. A single adult *F. candida* was released per Petri dish, and the plates were incubated at 21°C in darkness. The location of *F. candida* was observed and documented at specific time intervals: 0.5 h, 1 h, 2 h, 4 h, 8 h, 12 h, 24 h, 32 h, 48 h and 56 h after the release of the Collembola. A stereomicroscope (Zeiss Stemi 305) coupled with a Basler PowerPack pulse 5.0 microscope camera and the Basler Microscopy Software v1.2 were used to capture images and record the location of *F. candida*.

### 2.6. Dual-choice preference test

The preference of *F. candida* between wild-type *T. virens* Gv29.8 and each of its mutants was tested. Agar plugs (Ø = 9 mm) grown with *T. virens* strains were placed in the centre of a Petri dish (Ø = 92 mm) filled with moist plaster of Paris. A plug grown with the wild-type strain was placed on one edge of the Petri dish, while a plug with the respective mutant (knock-out or complement strain) was placed on the opposite edge. A single adult *F. candida* was released per Petri dish. For both strain combinations (wild-type versus knock-out and wild-type versus complement), a total of 42 replications in three rounds of experiments were performed. Plates were incubated at 21°C in darkness. Springtail location was assessed as described above. The distance of Collembola from the food sources was then measured using the software ImageJ v1.52a [35]. In the no-choice food acceptance test, Collembola were often observed in close proximity to the mycelium. For the dual-choice experiment, we defined attraction as a springtail being located within a radius of 10 mm from the centre of an agar plug, with at least 50% of its body within that radius. Collembola located more than 10 mm away from both agar plugs were counted as not having made a choice.

### 2.7. Fitness test

To obtain synchronized juvenile populations, small groups of adult sixth instar individuals were transferred to new plaster-charcoal plates, which triggers oviposition [36]. Newly laid eggs were gently transferred to another new plaster-charcoal plate and incubated at 20°C in darkness. The plates were checked daily to determine the hatching date of juveniles. Immature Collembola (10–12 days old) were transferred to new plaster-charcoal plates and fed with *T. virens* mycelium (wild-type, knock-out or complement respectively) on agar plugs (Ø = 9 mm) as the sole food source. Dry yeast was used as a positive control, and no food was given to the negative control. Plates were incubated at 21°C in darkness. Every three days, food was replaced, and tap water was added as needed to maintain humidity levels. Ten individuals were released per plate, and 10 replicates were performed for each treatment. The fitness test lasted 28 days. To investigate the survival rate, the number of surviving individuals was counted daily. Every 7 days, the plates were photographed using a Panasonic Lumix DMC-LX7 digital camera. Collembola body length (tail to head without antennae) was measured using the program Image J v1.52a. After 28 days, fecundity was measured by counting juveniles of the F_1_ generation.

### 2.8. Statistical analysis

Differences between strains in VOC and CO_2_ emission were analysed by a Kruskal-Wallis rank sum test [37] followed by a Dunn's test with Benjamini-Hochberg adjustment as a *post-hoc* comparison (*p* ≤ 0.05) [38].

For the olfactometer test, we analysed springtail preference between fungal strains using a generalized linear model (GLM) of the negative binomial family [39] followed by Tukey *post-hoc* comparison (*p* ≤ 0.05) [40] to investigate if springtail attraction was significantly different between the fungal strains. Location of Collembola were checked repeatedly during the course of the experiment and we analysed the sum of all Collembola choices to avoid temporal pseudo-replication. We also tried a generalized linear mixed effect model (GLMER) that included time point of observation as a fixed factor, but the random factors replicate identity (ID) and experimental round were singular in that model.

We analysed differences in acceptance of different fungal strains as a food source by *F. candida* with a GLM of the binomial family [37]. Acceptance was defined as described above. We created a data matrix from accepting Collembola and total tested Collembola across different time points and treatments using the cbind() function [37] and used it as the response variable. Time point of observation and fungal strain were included in the model for the acceptance test as fixed factors. Statistical differences between treatments were evaluated by factor level reduction analysis, where treatment levels were combined until model comparison revealed significant differences (ANOVA, *p* ≤ 0.05) in order to see how fungal strain influences the overall preference throughout the experiment [41].

For the dual-choice experiment, we used a GLMER of the binomial family [42] to investigate the differences in preference for wild-type or mutant strains between different strain combinations (wild-type versus knock-out and wild-type versus complement). Preference was defined as described above, and the time point of observation was included in the model as fixed factor, and replicate ID as a random factor. Differences in preference within treatment combinations of the dual-choice test were evaluated by two-sided *Z*-statistics (*p* ≤ 0.05) [37].

In order to analyse differences in *F. candida* survival when feeding on different food sources, we compared the proportion of dead *F. candida* after the animals had been exposed to the different diets for 28 days with a Kruskal-Wallis rank sum test [37] followed by Dunn's test with Bonferroni adjustment as a *post-hoc* comparison (*p* ≤ 0.05) [38] to determine significant differences between treatments.

To evaluate if the differences in sesquiterpene synthesis between the *T. virens* mutants lead to differential growth in *F. candida* feeding on them, we used a linear mixed effect model (LMER) [43] by comparing the average length of Collembola per replicate over time. Time point of observation and fungal strain were included in the model as fixed factors in interaction, replicate ID as a random factor. Statistical differences between treatments were evaluated by factor level reduction analysis, where treatment levels were combined until model comparison revealed significant differences (ANOVA, *p* ≤ 0.05) [41]. For this, we used the fixed factors without interaction between them.

To test whether reproduction of Collembola is affected by the lack of sesquiterpene formation, the average number of offspring per individual was calculated by dividing the number of juveniles per plate by number of adults that survived until the end of the experiment and the resulting average reproduction rate was analysed with a GLM of the negative binomial family [39] with Tukey *post-hoc* comparison between diet treatments (*p* ≤ 0.05) [40]. Fungal strain was included as a fixed factor.

All statistical analyses except the principal component analysis (PCA) were performed with R Studio version 1.2.1335 [37].

PCA was used to visualize and assess the volatile blends emitted by different *T. virens* genotypes in an unsupervised manner. Analysis was performed using MVSP 3.22 (Kovach Computing Services).

## 3. Results

### 3.1. Fungal volatile emission

**Figure 1.**
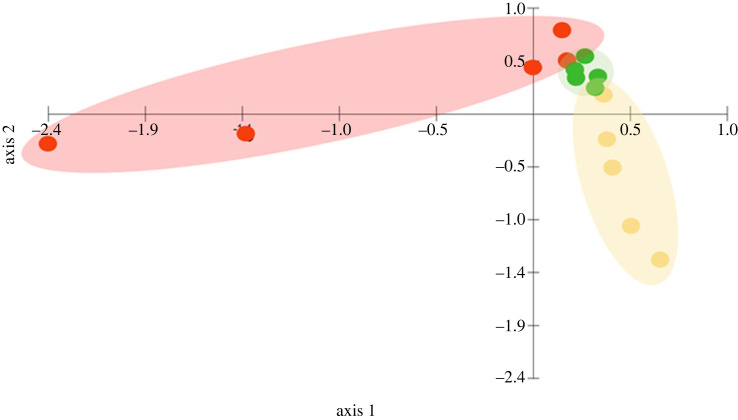
Principal component analysis of volatile organic compound emitted by *Trichoderma virens* Gv29.8 wild-type (red), knock-out mutant (green) and complementary (yellow) strains. Axis 1: PC 57.4%, axis 2: PC 30.51%.

We detected 22 sesquiterpenes and one monoterpene in the wild-type strain, the major compounds were ß-elemene, caryophyllene, γ-amorphene, γ-gurjunene, bicyclogermacrene, γ-cadinene and viridiflorol, which comprised 78.6% of the total emission (table 1). The knock-out strain produced only six sesquiterpenes in comparable amounts as the wild-type, notably dauca-4(11),8-diene (electronic supplementary material, figure S1A). Additionally, although in low amounts, the knock-out strain emitted two volatiles (germacrene D and an unidentified compound (RI = 1436.7)), which it only shares with the complement strain, but not the wild-type strain and emission rates of these two compounds did not differ between the two strains. In the complement strain, reintroduction of the *vir4* gene did not lead to full recovery of the VOC profile of the wild-type. Instead, three VOCs that were also produced by the wild-type and knock-out strains increased in quantity, while the two compounds it shares only with the knock-out do not differ significantly in quantity. Two sesquiterpenes were significantly elevated in the complement compared to the knock-out, but not the wild-type: calarenepoxide (Kruskal-Wallis: *Χ*^2^_2_ = 8.240, *p* = 0.016) and an unidentified sesquiterpene (RI = 1359.7) (Kruskal-Wallis: *Χ*^2^_2_ = 6.540, *p* = 0.04). Compared to wild-type and knock-out strain emissions, a significant increase was measured for the sesquiterpene daucene (Kruskal-Wallis: *Χ*^2^_2_ = 9.42, *p* = 0.01) (table 1). The two unique compounds observed in the complement strain were isodaucene and an unidentified sesquiterpene (RI = 1432.1) (electronic supplementary material, figure S1B). There were no statistical differences in emission between the three strains for dauca-4(11),8-diene. Significant differences were reported for 1,2,4a,5,6,8a-hexahydro-4,7-dimethyl-1-(1-methyl ethyl)-naphthalene (Kruskal-Wallis: *Χ*^2^_2_ = 6.66, *p* = 0.036), but these were not confirmed in the more conservative *post-hoc* Dunn's test. PCA analysis supports these results, displaying three distinct clusters for the strains, despite some overlaps (figure 1).

**Table 1.** Volatile profiles from *Trichoderma virens* Gv29.8 wild-type and mutants. (Lower case letters indicate significant differences in compound quantity between strains (*p* ≤ 0.05, Kruskal-Wallis rank sum test followed by Dunn's test with Benjamini-Hochberg adjustment as a *post-hoc* comparison). RI exp = experimental retention index, RI lit = retention index from literature, MT = monoterpene, ST = sesquiterpene, s.e. = standard error of the mean, * = compound identity confirmed by comparison with commercial standard.)

no.	class	compound	RI exp	RI ref	c(VOCs) (ng µl^−1^ eluate)					
					wild-type		knock-out		complement	
					average	s.e.	average	s.e.	average	s.e.
1	MT	β-myrcene*	992.9	991.0	0.6	0.3	—	—	—	—
2	ST	unidentified sesquiterpene	1359.7	—	0.7***^ab^***	0.1	0.4***^a^***	0.1	2.8***^b^***	0.8
3	ST	daucene	1391.1	1385.3	1.2***^a^***	0.2	1.1***^a^***	0.1	4.1***^b^***	1.0
4	ST	unidentified sesquiterpene	1395.3	—	0.3	0.2	—	—	—	—
5	ST	β-elemene	1402.3	1394.0	7.6	4.6	—	—	—	—
6	ST	α-gurjunene	1424.2	1424.0	0.7	0.5	—	—	—	—
7	ST	unidentified sesquiterpene	1429.5	—	0.5	0.3	—	—	—	—
8	ST	unidentified sesquiterpene	1432.1	—	—	—	—	—	0.4	0.2
9	ST	caryophyllene*	1435.6	1437.2	5.2	3.0	—	—	—	—
10	Other	unidentified compound	1436.7	—	—	—	1.4	0.5	1.3	0.6
11	ST	epi-β-caryophyllene	1459.9	1465.0	1.6	0.9	—	—	—	—
12	ST	unidentified sesquiterpene	1470.5	—	1.6	1.0	—	—	—	—
13	ST	germacrene D	1484.1	1484.0	—	—	0.3	0.0	1.2	0.4
14	ST	β-selinene	1487.7	1488.0	4.6	3.2	—	—	—	—
15	ST	γ-amorphene	1496.8	1496.0	16.1	9.4	—	—	—	—
16	ST	naphthalene, 1,2,4a,5,6,8a-hexahydro-4,7-dimethyl-1-(1-methylethyl)-	1501.2	—	1.1	0.7	1.0	0.3	4.9	1.0
17	ST	bicyclogermacrene	1512.7	—	6.2	3.8	—	—	—	—
18	ST	isodaucene	1516.0	1503.4	—	—	—	—	1.6	1.0
19	ST	γ-cadinene	1528.8	1525.0	14.0	6.9	—	—	—	—
20	ST	δ-cadinene	1535.6	1526.7	0.3	0.2	—	—	—	—
21	ST	unidentified sesquiterpene	1540.1	—	0.2	0.1	—	—	—	—
22	ST	dauca-4(11),8-diene	1545.8	1532.2	7.4	1.3	8.6	1.1	21.2	4.9
23	ST	calarenepoxide	1582.6	—	0.6***^ab^***	0.1	0.3***^a^***	0.1	3.7***^b^***	0.98
24	ST	palustrol	1587.6	1567.0	0.6	0.3	—	—	—	—
25	ST	carotol	1592.1	1594.0	0.1	0.1	—	—	—	—
26	ST	viridiflorol	1612.2	1609.0	12.2	7.1	—	—	—	—
27	ST	α-muurolol	1638.2	1639.0	0.4	0.2	—	—	—	—

Fungal CO_2_ emission was not significantly different between the strains (electronic supplementary material, figure S2).

### 3.2. Olfactometer tests

**Figure 2.**
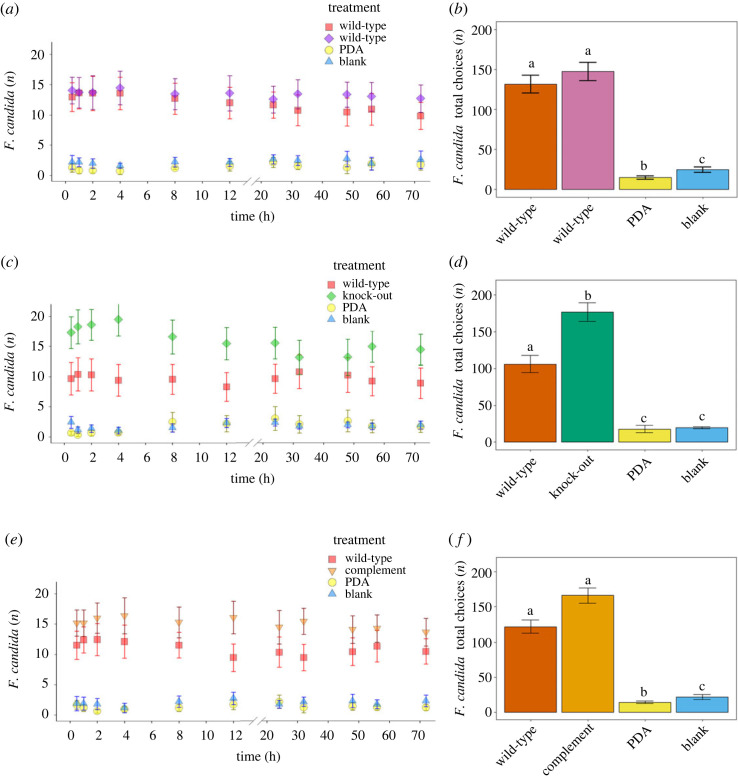
Olfactory orientation behaviour of *Folsomia candida* in response to volatiles of *Trichoderma virens* Gv29.8 and its mutants in a four-chamber olfactometer over a 72 h time period. Access to mycelium was denied by a filter paper walking arena. *n* = 30 individuals per olfactometer were released, and 24 olfactometers per treatment combination were evaluated. Line graphs show behavioural response of *F. candida* to mycelia over the duration of the experiment. Error bars indicate confidence interval (95%). Statistics were conducted on the sum of all choices within this time frame, depicted in bar graphs (*p* ≤ 0.05, generalized linear model with Tukey *post-hoc* analysis). Different letters indicate significant differences. Error bars indicate the standard error of the mean. (*a*) Collembola behaviour and (*b*) sum of choices with two times the wild-type strain as fungal volatile sources. (*c*) Collembola behaviour and (*d*) sum of choices wild-type and knock-out strains as fungal volatile sources. (*e*) Collembola behaviour and (*f*) sum of choices with wild-type and complement strains as fungal volatile sources.

Individuals of *F. candida* reacted strongly to the presence of fungal discs offered as odour sources in olfactometer tests. Significant differences in Collembola preference have been observed for all three tested strain combinations (wild-type versus wild-type: GLM: likelihood-ratio test (LRT)_3,92_ = 100.86, *p* < 0.001; wild-type versus knock-out: GLM*:* LRT_3,92_ = 102.40, *p* < 0.001; wild-type versus complement: GLM: LRT_3,92_ = 100.86, *p* < 0.001). *Folsomia candida* mostly ignored fungus-free PDA and empty compartments. When given a choice between two agar plugs grown with the wild-type, Collembola did not show a significant preference for either plug (Tukey: *Z* = 0.844, *p =* 0.833*, post-hoc* comparison; figure 2*a*,*b*), thus demonstrating that there was no bias for a specific side of the olfactometer. By contrast, significantly more Collembola congregated over the knock-out (Tukey: *Z =* 3.026, *p <* 0.013*, post-hoc* comparison; figure 2*c,d*) in comparison to the wild-type strain. More Collembola did also assemble over the complement than the wild-type strain, but the difference was not significant (Tukey: *Z =* 2.419 *p =* 0.073, *post-hoc* comparison; figure 2*e,f*).

### 3.3. No-choice and dual-choice acceptance test

**Figure 3.**
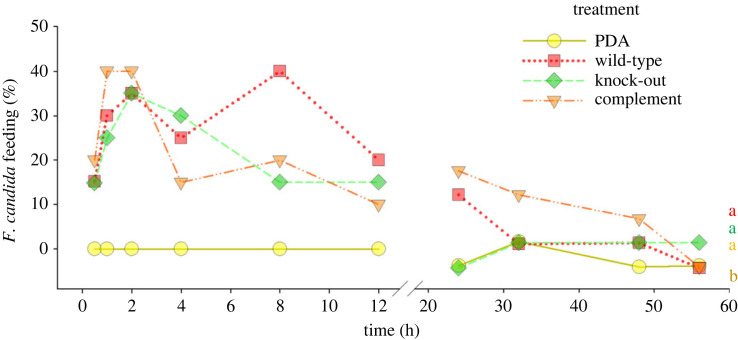
Acceptance of *Trichoderma virens* Gv29.8 strains as a food source by *Folsomia candida* in a no-choice test over 56 h. Acceptance was defined as *F. candida* being observed feeding on the mycelium. The response of 20 single individuals per treatment was observed. Lower case letters indicate statistical differences in food acceptance by *F. candida* between treatments throughout the experiment (*p* ≤ 0.05, generalized linear model with factor level reduction analysis).

Both treatment (GLM: *Χ*^2^_3_ = 48.824, *p* > 0.001) and time point (GLM: *Χ*^2^_1_ = 28.314, *p* > 0.001) had a significant effect on *F. candida* behaviour in the no-choice assay. Adult *F. candida* accepted all fungal strains as a food source with no significant differences. However, PDA without fungus was not attractive, and only one Collembola at one time point was observed feeding on it (figure 3).

**Figure 4.**
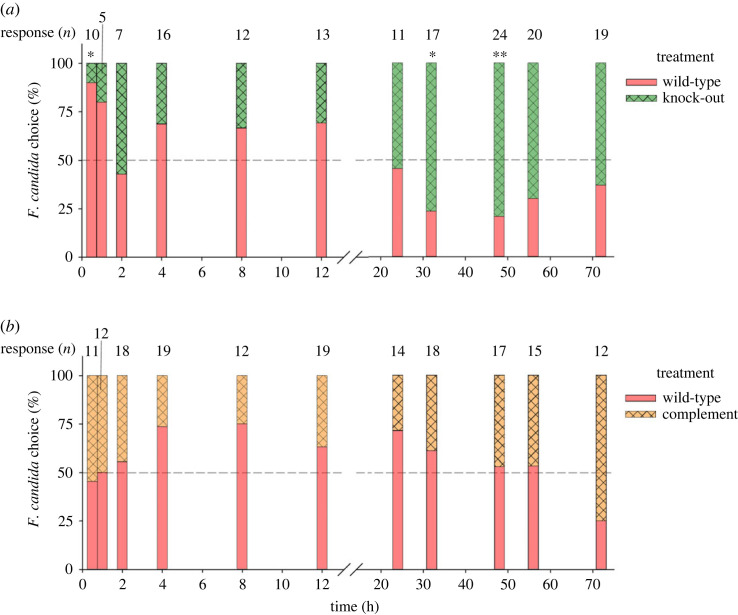
Food preference of *Folsomia candida* when offered mycelium of *Trichoderma virens* Gv29.8 and its mutants. (*a*) Wild-type versus knock-out (*b*) wild-type versus complement in a dual-choice test over 72 h as percentage of all responding individuals. Response was defined as location of an *F. candida* individual (*n* = 42 per combination) within a 10 mm radius from the centre of one of the mycelial plugs, and the number of responding individuals is indicated for each time point above the columns. No statistical differences in the ratio of preference between wild-type and mutant between the two treatment combinations has been detected (*p* = 0.157, generalized linear mixed effects model). Asterisks indicate significant differences in preference within a strain combination between wild-type and respective mutant strain (*p* ≤ 0.05, two-sided *Z*-test).

To determine whether springtails retain their preferences observed in the olfactometer test when provided access to the fungus, *F. candida* was allowed to choose between wild-type and knock-out strains or wild-type and complement strains of *T. virens* Gv29.8. We did not observe relevant differences in the choice behaviour of *F. candida* between the two treatment combinations wild-type versus knock-out (figure 4*a*) and wild-type versus complement (figure 4*b*) (GLMER: LRT = 1.715, *p* = 0.19). Attraction of *F. candida* varied over time (GLMER: LRT= 20.091, *p* < 0.001). *Folsomia candida* showed a significantly higher preference for the wild-type over the knock-out at the beginning of the experiment (0.5 h), while no significant preference was observed in the following 30 h. At 32 h and 48 h, *F. candida* significantly preferred the knock-out over the wild-type, but no preference was observed at later time points (figure 4). No significant preference between wild-type or complement could be observed at any time point.

### 3.4. Fitness test

**Figure 5.**
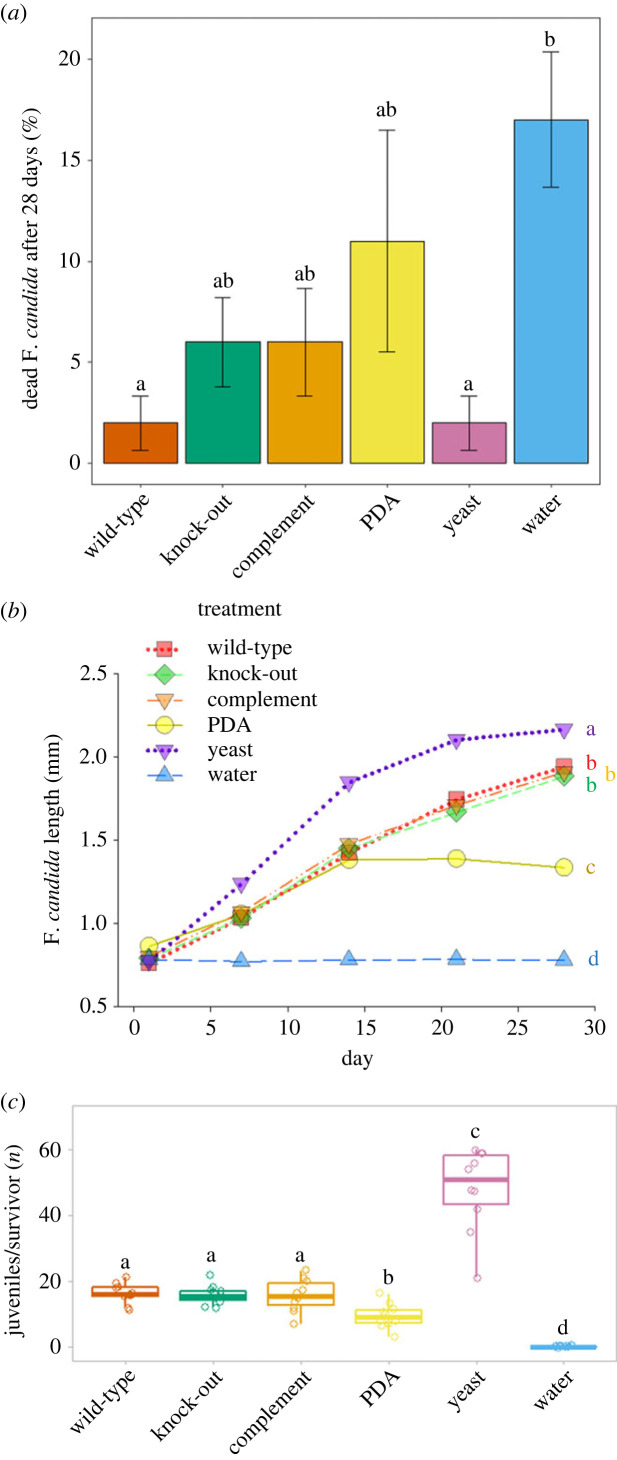
Fitness parameters measured for *Folsomia candida* in a 28-day fitness test under diets of *Trichoderma virens* Gv29.8 strains and control diets. A total of 100 individuals per treatment were observed in groups of 10. (*a*) Mortality of *F. candida* after 28 days. Whiskers indicate the standard error of the mean. Lowercase letters indicate statistical differences between treatments (*p* ≤ 0.05, generalized linear model with estimated marginal means *post-hoc* analysis). (*b*) Growth of juvenile *Folsomia candida*. Lowercase letters indicate statistical differences between treatments (*p* ≤ 0.05, linear mixed effects model with factor level reduction analysis). Error bars (standard error of the mean) were removed as they were so small that they were hardly visible. (*c*) Juveniles per surviving *Folsomia candida* from the parental generation. Whiskers extend to the most extended data point within 1.5*interquartile range. The upper box limit represents 3rd quartile, the lower box limit represents 1st quartile, and the middle box line represents 2nd quartile. Different letters above boxes indicate statistical differences between treatments (*p* ≤ 0.05, generalized linear model with Tukey *post-hoc* analysis).

Although diets had a significant impact on mortality (Kruskal-Wallis: *Χ*^2^_5_ = 17.714, *p* = 0.003), survival of Collembola was generally high. Even those groups that were provided only with water or PDA showed mortality of just 17% and 11%, respectively. Collembola that were provided with the fungal diet showed a lower mortality than those provided only with water. However, no significant differences were found between fungal strains, between fungal strains and the positive control, dried baker's yeast, or between fungal strains and the PDA control (figure 5*a*).

Diet (LMER: *F*_5,48_ = 345.35, *p* < 0.001), time (LMER: *F*_1,240_ = 2647.19, *p* < 0.001), and the interaction between these factors (LMER: *F*_5,240_ = 158.50, *p* < 0.001) had a significant influence on Collembola growth. Likewise, diet had a significant effect on fecundity of *F. candida* (GLMER: LRT_5,54_ = 47.15, *p* < 0.001). No significant differences in Collembola performance in reproduction and growth were observed between the fungal strains, but feeding on *T. virens* yielded lower growth and fecundity compared to feeding on yeast. Collembola feeding on PDA only performed significantly worse than Collembola feeding on *T. virens*. At the same time, the negative control group did neither grow nor reproduce (figure 5*b*,*c*).

## 4. Discussion

Our study suggests that fungal sesquiterpenes and the functionality of the terpene cyclase *vir4* in *T. virens* play an important role in the foraging behaviour of the fungivorous Collembola *F. candida.* Using olfactory cues alone to select fungal hyphae, *F. candida* was less attracted to the blend of VOCs emitted by wild-type *T. virens* Gv29.8 than to the sesquiterpene-reduced VOC profile of the Δ*vir4* knock-out strain. However, Collembola showed no significant preference when given a choice between wild-type and complement strains.

The VOC profiles of the wild-type, knock-out and complement strains differed considerably. In the knock-out mutant, most sesquiterpene VOCs were strongly reduced or absent. Reintroduction of *vir4* into the complement strain did not restore the phenotype of the wild-type strain, but increased the quantitative emission of some compounds. In addition, both the knock-out and complement strains produced novel VOCs, but not all of them were shared between them, in particular isodaucene, which was emitted exclusively by the complement strain. Visualization with PCA supports the assumption that the complement strain must be considered as a third unique VOC chemotype rather than an equivalent of wild-type *T. virens* Gv29.8.

Although we measured significant quantitative differences between strains in shared sesquiterpene compounds, the major difference between *T. virens* mutants appears to be qualitative, i.e. the absence of the compounds ß-elemene, caryophyllene, γ-amorphene, γ-gurjunene, bicyclogermacrene, γ-cadinene and viridiflorol, among others, in both the knock-out and complement strains. In our olfactometer bioassays, *F. candida* preferred the knock-out and complement strains when compared to *T. virens* Gv29.8 wild-type volatiles, but the preference was only significant for the knock-out strain, which emits the least sesquiterpenes. This implies that some of the VOC compounds which are absent from the odour of the knock-out and complement strains have a repellent effect on *F. candida*.

The sesquiterpenes daucene, dauca-4(11),8-diene, 1,2,4a,5,6,8a-hexahydro-4,7-dimethyl-1-(1-methylethyl)-naphthalene, and calarene epoxide were produced by all strains, including the knock-out, suggesting the existence of an alternative synthesis pathway for these sesquiterpenes in *T. virens*. Our results on volatile emission largely confirm previous studies on *T. virens* Gv.29.8. However, the compounds identified were not identical in every aspect owing to the different methods of volatile sampling employed and changes in growth media that may also influence VOC emission [29].

CO_2_ is emitted by many soil-dwelling organisms and is known to act as a general host cue for Collembola [16]. Our analysis showed that the fungal strains did not differ in CO_2_ emission, and thus CO_2_ emission could be excluded as an explanatory factor for the observed differences in olfactory behaviour.

Apart from the biosynthesis of sesquiterpene volatiles, other pathways in the secondary metabolism of *T. virens* Gv29.8, involving non-volatile compounds, are also affected by the deletion of *vir4* [44]. Non-volatile secondary metabolites can influence the gustatory food preferences of fungivores [7,45,46]. Collembola, for instance, prefer to graze on fungi with no apparent defence mechanisms and, to a lesser extent, on fungi such as *Piloderma croceum* or *Suillus luteus* that modify their hyphae to be physically unattractive by covering them with crystalline deposits [7]. Where possible, Collembola avoid feeding on fungi that produce repellent and toxic secondary metabolites [7]. Staaden *et al*. [14] demonstrated that Collembola can discriminate between fungi with impaired and intact chemical defences by olfaction alone, preferring the odour of defence-impaired *Aspergillus nidulans* colonies.

To investigate whether metabolic changes resulting from *vir4* deletion, other than in VOC emission, might affect the food preference of *F. candida*, we performed a series of bioassays. In no-choice tests, all *T. virens* strains were equally accepted as a food source. In dual-choice tests, which allow more subtle differences to be measured, *F. candida* initially preferred the wild-type strain and then switched its preference to the knock-out strain after 32 h. This was not the case when complement versus wild-type strains were offered. However, springtail preference between strain combinations was not statistically different in either dual-choice test with direct mycelial contact. Thus, while our results support the hypothesis that sesquiterpenes are an important factor influencing *F. candida* behaviour, direct access to the mycelium may relativize some of the VOC effects. This suggests additional effects of *vir4* deletion in *T. virens* on *F. candida* behaviour. The time-dependent shift in preference between wild-type and knock-out strain may be indicative of grazing-induced chemical defences [45,47], which may be regulated, at least in part, by *vir4*.

In *T. virens*, the terpene cyclase *vir4* is part of the *vir* gene cluster, which is also involved in the synthesis of the mycotoxins heptelidic acid, gliovirin and viridin. However, deletion of *vir4* does not affect the synthesis of these mycotoxins [48]. Therefore, sesquiterpene-mediated food preference is not related to the presence or absence of this class of secondary defence metabolites. We observed differences in pigmentation between *T. virens* Gv29.8 mutant strains (electronic supplementary material, figure S3). Although it is questionable how important visual cues are for soil-dwelling arthropods, pigmentation and/or gustatory cues from the pigments seem to play an important role in the food preference of Collembola. Many studies suggest that Collembola generally prefer feeding on dark-pigmented, melanised fungi [5,7,49], while others contest that these pigments are a decisive factor in food preference [50]. On the other hand, some pigments have been found to deter springtail grazing, like the bis-naphthopyrone aurofusarin [51] or the furocoumarin neurosporin A [52]. The identity of pigments and other secondary metabolites in *T. virens* that may affect Collembola food preference needs further investigation.

If the sesquiterpene emission of *T. virens* is coupled to *vir4*-dependent chemical defence, preferential feeding of *F. candida* on different *T. virens* strains should correlate with the fitness performance of the Collembola. Therefore, we fed juvenile *F. candida* on the mycelium of *T. virens* Gv29.8 mutant strains for 28 days to investigate the effect of *vir4* deletion on Collembola fitness. We did not find significant differences in mortality for *F. candida* feeding on different strains of *T. virens* Gv29.8. However, all fungal diets resulted in mortality below 10%, and even in the water control treatments without nutrients, survival after 28 days was very high. Furthermore, we did not observe significant differences in the fitness parameters growth and reproduction of *F. candida* when fed with the different strains. All strains allowed *F. candida* to grow and reproduce when used as the sole food source, but performance was always lower than the positive control, baker's yeast.

Previous studies have shown that *Trichoderma* species are generally unattractive to fungivores such as Collembola and are therefore presumably well defended. Severe fitness penalties, including the failure to reproduce, have been reported for the Collembola *Onychiurus sinensis* when feeding on *Trichoderma polysporum* [21] and for *F. candida* and *Folsomia fimetari* when feeding on *Trichoderma harzianum* [20]. Klironomos & Ursic [19] showed that *F. candida* feeding on *T. harzianum* resulted in intermediate reproductive performance and survival compared to two other fungi, suggesting strong species- and strain-specific effects. Fungal toxins have been suggested to cause reduced survival and reproduction in Collembola when feeding on *Trichoderma* spp., but no evidence has yet been provided to support this hypothesis. These effects on springtail fitness coincided with a general avoidance of *Trichoderma* strains when other saprophytic or pathogenic fungi were available as food sources [19–22]. By contrast, *F. candida* and *Protaphorura armata* showed high fitness and preference when feeding on an unidentified *Trichoderma* sp. [50].

In the case of *T. virens* Gv29.8, the food preference of *F. candida* was not linked to differences in fitness for this fungivore. Host preferences of fungivores do not always correspond to optimal fitness gains, and food sources that are less preferred in preference tests might not have consequences for fungivore fitness [50,53].

Our understanding of the biological functions of fungal sesquiterpenes in the interactions with arthropods is still incomplete. Much more is known from plants, where sesquiterpenes play multiple ecological roles concerning plant reproduction and defence. In plants, sesquiterpenes are involved in attracting pollinators [54] and natural enemies of herbivores [55,56], or may act as natural defences against herbivores [57,58]. Analogous functions have been hypothesised for fungal sesquiterpenes, but experimental evidence for this is scarce [31]. Sesquiterpenes are generally produced in the late stages of fungal growth. Becher *et al*. [15] showed that the sesquiterpene geosmin attracts *F. candida* to the hyphae-forming bacterium *Streptomyces coelicolor*. Geosmin synthesis is co-regulated with sporulation by the response regulator transcription factor *BldM*, suggesting a targeted attraction of fungivores to aid in reproduction, analogous to floral scent in plants. To the best of our knowledge, this is the only study to date that has ascribed an important biological function to a volatile sesquiterpene in springtails. Similarly, fungivorous beetles are attracted by fungal sesquiterpenes, as they use these compounds for host location [33]. By contrast, our findings support a defence-related function for sesquiterpenes, which has also been found for microbial sesquiterpenes acting on soil-dwelling nematodes [59].

In conclusion, we demonstrate that sesquiterpene VOCs produced by *T. virens* were repellent but not toxic to *F. candida.* Neither sesquiterpenes nor other metabolic changes had significant effects on fungivore survival, growth, and reproduction. Our data also support the role of olfactory cues as an important factor in Collembola food location and preference [14–18,60]. Besides the direct consequences of *vir4* deletion, changes in the induced defence against fungivore grazing may be the reason for the increased Collembola attraction towards the knock-out strain. Fungivore grazing can shape competition between fungal species [2–6], while the biocontrol activity by *Trichoderma* is often based on outcompeting pathogenic strains [23,24]. Our research may serve as a first step in understanding how grazing fungivores affect the efficacy of *Trichoderma* strains and how their secondary metabolism influences these interactions.

## Data Availability

The data are provided at doi:10.25625/8LZP1H [61] and also in the electronic supplementary material [62].
